# Nanotoxic Profiling of Novel Iron Oxide Nanoparticles Functionalized with Perchloric Acid and SiPEG as a Radiographic Contrast Medium

**DOI:** 10.1155/2015/183525

**Published:** 2015-05-17

**Authors:** Muhamad Idham Mohamed, Mohd Khairul Amran Mohammad, Hairil Rashmizal Abdul Razak, Khairunisak Abdul Razak, Wan Mazlina Md Saad

**Affiliations:** ^1^Department of Medical Laboratory Technology, Faculty of Health Sciences, Universiti Teknologi MARA, UiTM Puncak Alam, 42300 Selangor, Malaysia; ^2^Department of Medical Imaging, Faculty of Health Sciences, Universiti Teknologi MARA, UiTM Puncak Alam, 42300 Selangor, Malaysia; ^3^NanoBiotechnology Research and Innovation, Institute for Molecular Medicine, Universiti Sains Malaysia (USM), 11800 Penang, Malaysia

## Abstract

Emerging syntheses and findings of new metallic nanoparticles (MNPs) have become an important aspect in various fields including diagnostic imaging. To date, iodine has been utilized as a radiographic contrast medium. However, the raise concern of iodine threats on iodine-intolerance patient has led to search of new contrast media with lower toxic level. In this animal modeling study, 14 nm iron oxide nanoparticles (IONPs) with silane-polyethylene glycol (SiPEG) and perchloric acid have been assessed for toxicity level as compared to conventional iodine. The nanotoxicity of IONPs was evaluated in liver biochemistry, reactive oxygen species production (ROS), lipid peroxidation mechanism, and ultrastructural evaluation using transmission electron microscope (TEM). The hematological analysis and liver function test (LFT) revealed that most of the liver enzymes were significantly higher in iodine-administered group as compared to those in normal and IONPs groups (*P* < 0.05). ROS production assay and lipid peroxidation indicator, malondialdehyde (MDA), also showed significant reductions in comparison with iodine group (*P* < 0.05). TEM evaluation yielded the aberration of nucleus structure of iodine-administered group as compared to those in control and IONPs groups. This study has demonstrated the less toxic properties of IONPs and it may postulate that IONPs are safe to be applied as radiographic contrast medium.

## 1. Introduction

Iodine contrast medium has long been applied in CT scanning for* in vivo* imaging; however some problems may arise such as short imaging time and high toxicity to kidney [1, 2]. In order to overcome this drawback, a contrast agent which demonstrates lower toxicity level has been searched thoroughly and metallic nanoparticles (MNPs) such as iron oxide nanoparticles (IONPs) have offered their potential superiority [2, 3]. Nanotechnology is the branch of science which deals with modification and synthesization of materials in nanometer size. A lot of scientific researches focus on MNPs due to their unique properties which are beneficial in various fields [4]. Current nanomedicine has utilized MNPs as novel mediator in targeting drug therapy and biomedical imaging [5]. MNPs have been shown to be a good contrast agent and can improve limitations of conventional iodine such as longer acquisition time and lower toxicity [1, 6].

IONPs have been thoroughly studied in medical imaging and several types are very convincing due to their biocompatibility properties such as magnetite (Fe_3_O_4_) and haematite (*α*-Fe_2_O_3_) [7]. However, it has been reported that IONPs could induce oxidative stress [8]. Peng et al. [9] revealed that IONPs have a long retention time in circulation and biodegradable and lower toxicity. Moreover, polyethylene glycol (PEG) derivatives are usually used for coating since it can minimize opsonization of IONPs. Brullot et al. expressed that silane-PEG (SiPEG) acts as stabilizer which is soluble in both polar and nonpolar solvents, and thus it will be inert and biocompatible [10]. Concerning the use of MNPs, the uncertainty of the toxicity criteria requires full scale analysis and risk assessment. To ensure the nanotechnology development is beneficial, various toxicity characteristics of metallic NPs have been reported [11].

Basic concept of MNPs toxicity may be explained by the production of reactive oxygen species (ROS) which triggers oxidative stress. This phenomenon is considered as the main underlying process of nanotoxicology [11, 12]. An* in vitro* toxicological study by Zhao et al. [13] has demonstrated that MNPs can trigger ROS formation [14]. Usually, the nanometal or metal oxides may enhance ROS to induce oxidative stress and DNA damage which may lead to apoptosis or carcinogenesis. However, the shape is not a main critical determinant of nanotoxicity. Previous research suggested that toxicity of MNPs was mediated by lipid peroxidation, ROS, and oxidative stress [15]. For century, biochemical and cell-based method have been used in assessing the nanotoxicity due to the benefits of cell line variety and reproducibility. However, the measurement of* in vivo* mechanism is rather complex and challenging, as it may represent the actual environment of homeostasis [11]. Understanding the MNPs behavior will be the key answer towards interpretation of toxicological results [16].

In the past, many MNPs for biomedical applications had been studied for their toxicology aspects via* in vitro* and* in vivo* investigations. The interaction between exogenous MNPs and serum proteins after entering circulation may reveal that they were transported in some tissues such as liver and caused hepatotoxicity [17]. In some cases, intravenous administration of MNPs distributes mainly in liver and retains without indicating systemic toxicity [18, 19], whereas Cho et al. reported that oral ingestion of zinc oxide NPs was deposited mainly in liver and kidney within 72 hours of their administration [20]. Thus, the present study aimed to provide a scientific evaluation of* in vivo* IONPs nanotoxicity in the liver following the implementation as a contrast medium.

## 2. Materials and Methods

### 2.1. Chemicals

IONPs were obtained from NanoBiotechnology Research and Innovation, Institute for Research in Molecular Medicine (NanoBRI @ INFORMM), Universiti Sains Malaysia, Malaysia. Conventional iodinated contrast medium was purchased from GE Healthcare, Malaysia. Oxiselect TBARS Assay Kit (MDA Quantification) and Oxiselect* In Vitro* ROS/RNS Assay Kit (Green Fluorescence) were purchased from Cell Biolab, Inc. (San Diego, CA); other chemicals were purchased from Merck Company.

### 2.2. Particles Characterization

The particles size and shape were determined by using transmission electron microscope (TEM). In brief, MNPs solution was sonicated for 10 minutes. One drop was deposited onto copper grid TEM and allowed to dry in air. NPs solution was again sonicated for 10 minutes and one drop of solution was placed on copper grid for viewing process by FEI TECHNAI G2.

### 2.3. Animals and Treatment

Animal study was conducted in accordance with the guidelines of Universiti Teknologi MARA Committee of Animal Research and Ethics (UiTM CARE) concerning the use of experimental animals (Ref: 28/2013). Fifteen of healthy four-week-old Wistar rats weighing about 200 grams were obtained from Laboratory Animal Facility and Management (LAFAM), UiTM Puncak Alam Campus. The animals were acclimatized for two weeks. Normal pellet diet with filtered water was given* ad libitum*. Experiments were performed on healthy six-week-old Wistar rats weighing about 250 grams. The study consisted of three groups (*n* = 5) of six-week-old Wistar rats which are divided into control group (Cx), iodine group (Ix), and iron oxide nanoparticles group (IONPx). Animal from Ix and IONPx received 0.5 mL of 300 *μ*g/mL commercial iodine and IONP* via* intravenous administration. After 24 hours, blood sample was collected in ethylenediaminetetraacetic acid (EDTA) and Plain tube from orbital venous plexus under slight diethyl ether anesthesia. All of the animals were sacrificed by cervical dislocation after 24 hours. Liver tissues were excised immediately and stored at −80°C prior to further analysis.

### 2.4. Hematological and Biochemistry Analysis

Whole blood sample was sent for hematological analysis and the serum was collected after centrifugation of Plain tube at 10,000 g for 15 minutes. Total red blood cells (TRBC), white blood cells (TWBC), and platelet count (PC) were performed for hematology parameters. Meanwhile for biochemistry assessment, alanine transaminase (ALT), aspartate aminotransferase (AST), and alkaline phosphatase (ALP) were performed. The assessment of hematology and biochemistry parameters was done in University Veterinary Hospital, UPM Serdang. Hematology and biochemistry analyses were conducted according to Shahbazi et al. [21].

### 2.5. Measurements of Cellular Reactive Oxygen Species (ROS)

The liver cellular ROS generation level was estimated by the method of Oxiselect* In Vitro* ROS Assay (Green Fluorescence). Marquis et al. [22] stressed that studies on ROS produced from* in vitro* NPs exposure have been extensively conducted, and DCFDA is amongst widely used methods in nanotoxicology. Tissue samples were resuspended at 50 mg/mL in PBS and homogenized on ice. Sample was spun at 10,000 g for five minutes. Supernatant was collected and assayed directly for ROS production determination. Oxidation reaction of ROS samples with DCFH probe was measured fluorometrically at 480 nm excitation/530 nm emission with POLARstar Omega Plate Reader. Free radical content was determined by comparison with the predetermined DCF standard curve.

### 2.6. Lipid Peroxidation Product, MDA Assay

Lipid peroxidation of liver was determined as the concentration of malondialdehyde (MDA) generated by the thiobarbituric acid (TBA) reaction by Guo et al. [23]. Tissue samples were resuspended at 100 mg/mL in PBS containing 1X butylated hydroxytoluene (BHT) and homogenized on ice. The sample was spun at 10,000 g for five min. Supernatant was collected and assayed directly for TBARS level. MDA in samples and standards interacted with TBA at 95°C and the samples were incubated and then measured spectrophotometrically at 532 nm with POLARstar Omega Reader. MDA level was determined by comparison with predetermined MDA standard curve.

### 2.7. Observation of Liver Ultrastructure

In measuring the cellular and biochemical alterations pertaining to NPs administration, a variety of microscopic evaluations has been described by Schrand et al. [24]. Tissue samples were placed in 4% glutaraldehyde immediately after excision into 1 mm × 1 mm size for fixation. Samples were then washed with sodium cacodylate buffer and postfixed with 1% osmium tetroxide in 0.1 M cacodylate buffer. Osmicated samples were dehydrated with graded series of ethanol (50%, 70%, 95%, and 100%) and rinsed in propylene oxide before being embedded in resin mould. Resin blocks were trimmed and proceed to semithin sectioning on glass slide then stained with toluidine blue for characterization of cells using light microscope. After the semithin sectioning was done, the ultrathin sectioning was cut and mounted on copper grid. Grids were contrasted with uranyl acetate and lead citrate for ultrastructural evaluation by using TEM (FEI TECHNAI G2).

### 2.8. Statistical Analysis

Statistical analysis was performed by SPSS version 18.0 (SPSS Inc., Chicago, IL, USA) for Windows. Data were analyzed by one-way analysis of variance (ANOVA) and followed by post-hoc Tukey test for multiple comparison of mean. A *P* value < 0.05 was considered as statistically significant.

## 3. Results

### 3.1. NPs Size and Shape Characterization

**Figure 1:**
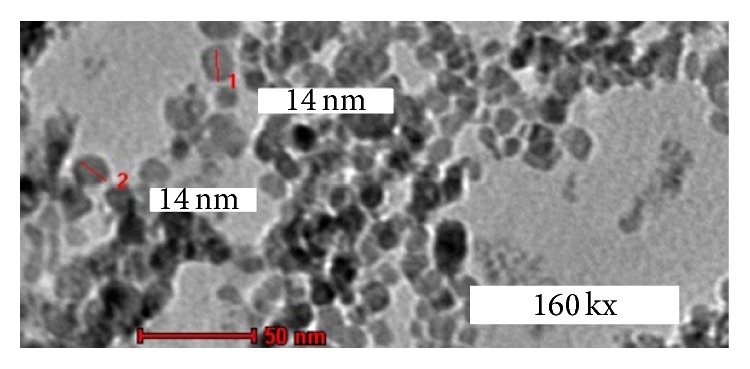
IONPs microstructure. 1 and 2: the diameter of the IONPs. Spheres of IONPs depicted in uranyl acetate and lead citrate contrast. The average of IONPs size is 14 nm.

The characterization was carried out using TEM (FEI TECHNAI G2) with 160,000 times (160KX) magnification showing the size and shape of IONPs. The IONPs are spheres in shape with “grapelike” arrangements and the overall diameter is 14 nm approximately (Figure 1).

### 3.2. General Examination

After administration of iodine and IONPs, the animals were closely monitored for lethal sign or any obvious physical changes. No mortality or any significant clinical sign was observed. Gross examination of the liver for both particles-administered animals also reveals no evidence of pathological changes.

### 3.3. Hematological Analysis

**Figure 2:**
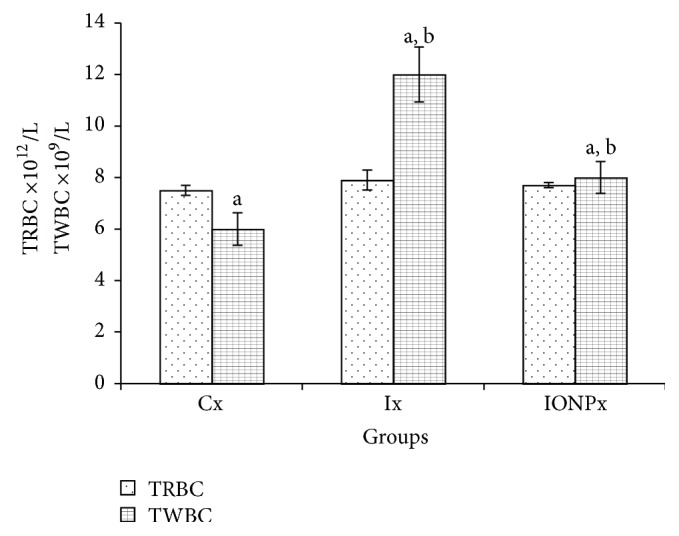
Hematology values. The bar chart shows the values of TRBC and TWBC in Cx, Ix, and IONPx groups. Values were expressed as mean ± S.E.M (*n* = 5)(*P* < 0.05). ^a^Significant differences when compared to Cx, (*P* < 0.05). ^b^Significant differences when compared to Ix (*P* < 0.05).

**Figure 3:**
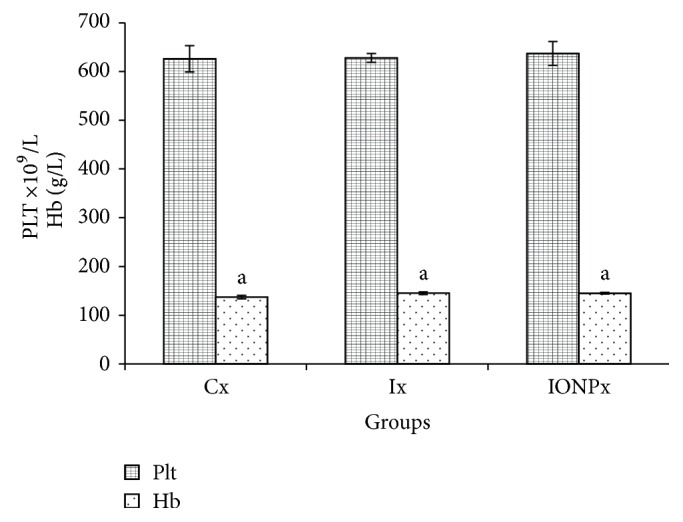
Platelets and hemoglobin values. The bar chart shows the values of PLT and Hb in Cx, Ix, and IONPx groups. Values were expressed as mean ± S.E.M (*n* = 5)(*P* < 0.05). ^a^Significant differences when compared to Cx (*P* < 0.05).

Hematological parameters include total red blood cells (TRBC), total white blood cells (TWBC), platelet (PLT), and hemoglobin (Hb). The levels of TRBC and WBC are shown in Figure 2, while the levels of PLT and Hb are shown in Figure 3. TRBC level was in normal range and no significant differences were observed between Cx and both treatment groups (Figure 2). In contrast, the TWBC for Ix has shown a significant increment compared to Cx and IONPx (*P* < 0.05), respectively. The value for Ix increased almost twofold from Cx. Value of IONPx also increased compared to Cx (*P* < 0.05). On the other hand, the PLT levels did not show any significant differences in all groups. Figure 3 also presents that the level of Hb for both Ix (145.2 g/L) and IONPx (145 g/L) was significantly higher compared to Cx (137.2 g/L) (*P* < 0.05), respectively.

### 3.4. Biochemistry Analysis

**Figure 4:**
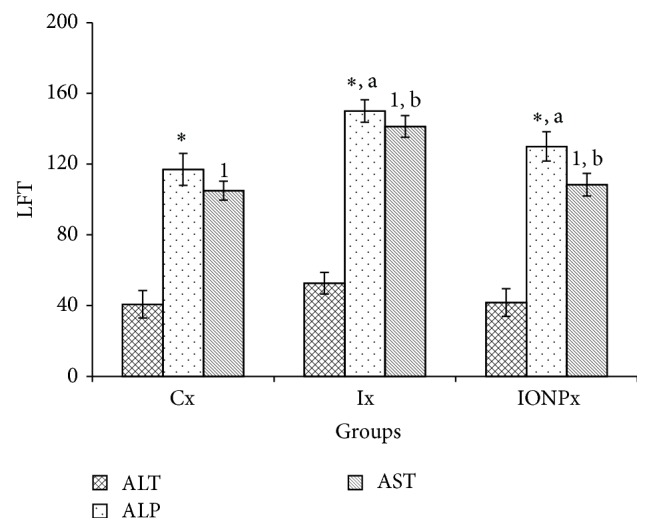
Liver biochemistry values. The chart line shows the values of ALT, ALP, and AST in Cx, Ix, and IONPx groups. Values were expressed as mean ± S.E.M (*n* = 5)(*P* < 0.05). ^*^Significant differences when compared to Cx (*P* < 0.05). ^a^Significant differences when compared to Ix (*P* < 0.05). ^1^Significant differences when compared to Cx (*P* < 0.05). ^b^Significant differences when compared to Ix (*P* < 0.05).

The effects of intravenous administrations of 300 *μ*g/mL of iodine and IONPs were evaluated through the measurement of different biochemical parameters. In this study, Ix ALP level was significantly increased compared to Cx (*P* < 0.05) and IONPx (*P* < 0.05) (Figure 4). No significant differences were observed between all groups in the ALT levels. Level of AST, on the other hand, showed a significant increment in Ix compared to Cx and IONPx (*P* < 0.05) (*P* < 0.05), respectively.

### 3.5. ROS Assay

**Figure 5:**
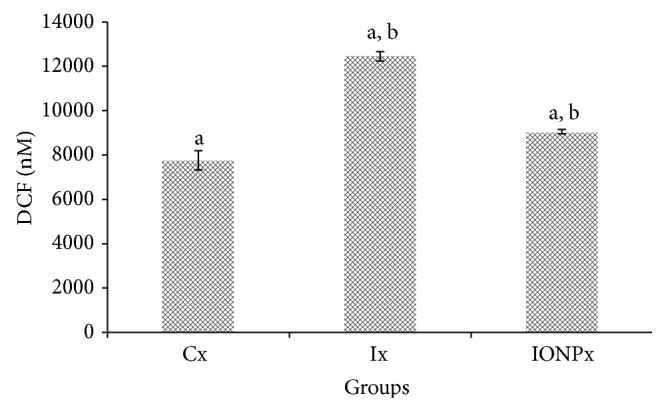
ROS production values. The chart shows the values of ROS production in liver's tissues of Cx, Ix, and IONPx groups. Values were expressed as mean ± S.E.M (*n* = 5)(*P* < 0.05). ^a^Significant differences when compared to Cx (*P* < 0.05). ^b^Significant differences when compared to Ix (*P* < 0.05).

The determination of ROS in liver after being administered with particular particles has been done and the results were described in Figure 5. Liver ROS production in Ix was significantly increased compared to Cx and IONPx (*P* < 0.05). Significant increment of liver ROS level was also observed in IONPx compared to Cx (*P* < 0.05).

### 3.6. MDA Assay

**Figure 6:**
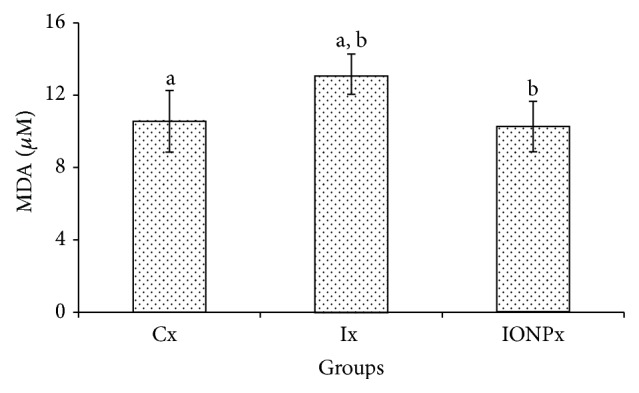
MDA values. The bar chart shows the values of MDA level in liver's tissue of Cx, Ix, and IONPx groups. Values were expressed as mean ± S.E.M (*n* = 5)(*P* < 0.05). ^a^Significant differences when compared to Cx (*P* < 0.05). ^b^Significant differences when compared to Ix (*P* < 0.05).

Lipid peroxidation level in liver was assessed by the determination of its major end product, malondialdehyde (MDA). The quantification of MDA would reflect the level of lipid peroxidation occurrence in liver.  Figure 6 demonstrated that liver's MDA was significantly higher in Ix compared to Cx and IONPx (*P* < 0.05), respectively. The level of MDA in IONPx was lower than the value of Cx; however no significant difference had been noted.

### 3.7. Observation of Liver Ultrastructure

**Figure 7:**
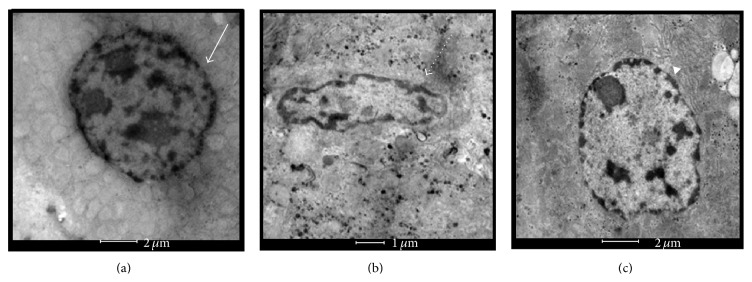
Ultrastructure of nucleus in liver. (a) Cx nucleus (straight line); (b) Ix nucleus (dotted line); (c) IONPx nucleus (arrow head pointed figures). Cx and IONPx nucleus are with smooth membrane outline and regular in size, while Ix nucleus reduced in size. Ix is also noted to be irregular and dense nucleus membrane outline.

The ultrastructural observation of the liver's nucleus had been carried out by using TEM and the results were depicted in Figures 7(a), 7(b), and 7(c). Structure of nucleus membrane from Ix liver tissues (Figure 7(b)) shows an irregular outline compared to the nucleus membranes in Cx (Figure 7(a)) and IONPx (Figure 7(c)). The shrinkage in size was also observed in Ix nucleus when the comparison was made with Cx and IONPx. Nucleus outline of Ix also was noted to be denser than Cx and IONPx (arrow pointed figures).

## 4. Discussion

Nanotechnology has emerged rapidly due to its various functions and among the most important benefit of nanotechnology is in biomedical application. However, the toxicological impact for the body should be thoroughly identified as they may cause unfavorable effects due to interaction with body components [25]. Liver is a major organ where the particles are deposited and inducing damage [15, 26]. The accumulation of nanoparticles in the liver may be due to Kupffer cells intake for detoxification process [26]. In this study, we evaluate toxicological effects on rat's liver after intravenous injection of IONPs by complete blood count (CBC/FBC), liver function test (LFT), ROS production, lipid peroxidation, and ultrastructural evaluation.

A CBC was performed to assess the blood components environment and to detect any pathological changes in homeostasis. Selected parameters consisted of TRBC, TWBC, PLT, and Hb which had been done as they are pathologically relevant to the hematotoxicity study. TRBC shows no significant differences in IONPx, Ix, and Cx. This may indicate no abnormalities in red blood cells and no anemic symptoms. The administration of IONPx did not induce red cells defect and this was supported by the findings of Ahamed et al., which point out that MNPs did not induce red blood cells defect [15]. On the contrary, the level of TWBC in Ix shows a significant increment compared to Cx and IONPx. This phenomenon is known as leucocytosis, where the study by Dobrovolskaia and McNeil found that leucocytes proliferation may indicate that the foreign substances carry immunostimulatory properties [27]. Level of PLT on the other hand shows no significant differences between all groups which indicates normal level of platelet, and this was also observed from the previous study [15]. A level of Hb in Ix and IONPx was found to be significantly increased compared to Cx, but the level was in normal range. This was supported by Ahamed et al., who expressed that NPs administration did not induce significant alteration in hematologic parameters [15] and Marquis et al. [22] explained that alteration of FBC level would reflect the occurrence of toxicity.

In LFT, the level of ALP and AST in Ix was significantly elevated when comparison had been made with Cx and IONPx. This may indicate that iodine has led to a distortion in liver function while the IONPs did not induce any significant changes to the liver. This study's findings are in agreement with García et al., which noted that the IONPs did not induce toxicity to the experimental animals and can be considered safe to be used [28]. Other findings by Guo et al. [23] and Ahamed et al. described similar scenario, in which the level of serum biochemistry in NPs administered group did not indicate changes compared to control group [15]. The level of ALT on the other hand did not show differences between all groups but the values were higher in Ix compared to both Cx and IONPx. van der Zande et al. also expressed the same phenomenon that, in nanosilica-treated animal, the blood biochemistry showed no liver intoxication [18].

Excess reactive oxygen species (ROS) may induce oxidative stress in cellular environment by the imbalance of redox status and can lead to various pathological disorders [29]. The evaluation of ROS production may provide useful information regarding the capability of certain substances or environments in inducing oxidative stress. Whenever oxidative status is in imbalance, it may induce toxicological effect [22]. The level of ROS in Ix was significantly higher compared to Cx and IONPx. Meanwhile, the level of free radicals in IONPx also showed an elevation when comparison was made with Cx. These results may explain that the administration of NPs and iodine both induces ROS production in the liver tissues. However, the free radicals that produced in Ix were significantly higher than those of IONPx and thus may indicate that IONPs produced lower ROS compared to iodine. Ahamed et al. mentioned that ROS generation and oxidative stress are likely to induce toxicity of NPs [15]. Auffan et al. [30] further explained that excess ROS may lead to a toxic potential of NPs. However, considering the level of ROS in both Ix and IONPx, this study may suggest that the administration of IONPs shows fewer tendencies to induce toxicity compared to iodine. This was further supported by Roy et al., who point out that the enhancement of ROS generation will lead to toxicity [26]. Fu et al. addressed that nanomaterial-induced ROS is essential to determine nanotoxicity [11]. In addition, this present study was in line with Chairuangkitti et al., who revealed that the ROS level was related to intracellular ROS generation [31].

Lipid peroxidation is considered as one of the mechanisms involved in oxidative damage prior to excess ROS production [26], and the main byproduct is malondialdehyde (MDA). Measuring the level of MDA would reflect that a lipid peroxidation mechanism occurred. Fu et al. expressed that IONPs exhibit low to no toxicity [11]. The level of MDA in Ix was significantly increased compared to Cx and IONPx while no significant values were found to be detected between Cx and IONPx. This indicates that iodine has led to a lipid peroxidation in liver tissues while IONPs did not induce membrane lipid damage in liver of IONPs administered rats. The findings were in agreement with ROS generation which showed that the level of ROS in IONPx was significantly lower than in Ix. The outcomes of this study were in agreement with previous research done by [32], which expressed that the NPs administration did not lead to significant changes in liver's MDA level.

Ultrastructural evaluation by TEM revealed that IONPs administration did not induce any significant pathological damage when a comparison was made to control group. The micrograph of Ix however shows a slight aberrant shape of nucleus with shrinking in size and thickening of membranes with irregular nuclear membrane. The ultrastructural evaluation results are in agreement with the other parameter findings, which reveal that there is no significant deterioration of liver tissues in IONPs administered Wistar rats compared to iodine. A study by Li et al. [17] stated that superparamagnetic IONPs will only trigger the toxicity in high dose with repeated injection while Brullot et al. point out that IONPs with SiPEG are biocompatible [10]. According to this study, IONPs show less toxic properties compared to iodine and are in good agreement with other research findings.

This present study involved* in vivo* nanotoxicity and did not cover* in vitro* study due to limited timeframe, budget, and facilities. However, the limitation has been encountered by using a systematic study and depicts an actual body mechanism. Future research should focus on molecular level toxicity assessment of iodine and IONPs for better understanding.

## 5. Conclusion

The present study has addressed the* in vivo* nanotoxicity profile for IONPs in radiographic applications and the results might suggest that IONPs are safe to be applied as a contrast medium.
